# Maternal Geohelminth Infections Are Associated with an Increased Susceptibility to Geohelminth Infection in Children: A Case-Control Study

**DOI:** 10.1371/journal.pntd.0001753

**Published:** 2012-07-24

**Authors:** Raaj S. Mehta, Alejandro Rodriguez, Martha Chico, Irene Guadalupe, Nely Broncano, Carlos Sandoval, Fernanda Tupiza, Edward Mitre, Philip J. Cooper

**Affiliations:** 1 Laboratorio de Investigaciones, Fundacion Ecuatoriana Para la Investigacion en Salud (FEPIS), Quinindé, Esmeraldas Province, Ecuador; 2 Hospital “Padre Alberto Buffoni,” Quinindé, Esmeraldas Province, Ecuador; 3 Colegio de Ciencias de la Salud, Universidad San Francisco de Quito, Quito, Ecuador; 4 Uniformed Services University of the Health Sciences, Bethesda, Maryland, United States of America; 5 Molecular and Biochemical Parasitology, Liverpool School of Tropical Medicine, Liverpool, United Kingdom; Case Western Reserve University School of Medicine, United States of America

## Abstract

**Background:**

Children of mothers infected with soil-transmitted helminths (STH) may have an increased susceptibility to STH infection.

**Methods and Findings:**

We did a case-control study nested in a birth cohort in Ecuador. Data from 1,004 children aged 7 months to 3 years were analyzed. Cases were defined as children with *Ascaris lumbricoides* and/or *Trichuris trichiura*, controls without. Exposure was defined as maternal infection with *A. lumbricoides* and/or *T. trichiura*, detected during the third trimester of pregnancy. The analysis was restricted to households with a documented infection to control for infection risk. Children of mothers with STH infections had a greater risk of infection compared to children of uninfected mothers (adjusted OR 2.61, 95% CI: 1.88–3.63, p<0.001). This effect was particularly strong in children of mothers with both STH infections (adjusted OR: 5.91, 95% CI: 3.55–9.81, p<0.001). Newborns of infected mothers had greater levels of plasma IL-10 than those of uninfected mothers (p = 0.033), and there was evidence that cord blood IL-10 was increased among newborns who became infected later in childhood (p = 0.060).

**Conclusion:**

Our data suggest that maternal STH infections increase susceptibility to infection during early childhood, an effect that was associated with elevated IL-10 in cord plasma.

## Author Summary

Soil-transmitted helminths (intestinal worms) are among the most common childhood infections worldwide and are a significant cause of morbidity particularly among poor populations living in developing countries. The potent immune modulatory effects of these parasites have been suggested to be a determinant of the epidemiological distributions of other infectious diseases (e.g., HIV and tuberculosis) and allergy. There is strong epidemiological evidence that some individuals have an increased susceptibility to re-infection after treatment and the mechanisms underlying this are not well understood. A possible explanation is that *in utero* exposure to maternal STH infections may increase the risk of infection during childhood, but, as far as we are aware, no published study has addressed this hypothesis for STH infections in humans. In this study, we evaluated whether children of mothers infected with STH infections have a greater risk of infection when compared to children of uninfected mothers. We also examined whether this increased susceptibility to infection might occur through the tolerogenic effects of increased levels in the systemic circulation of the immune regulatory cytokine IL-10, in early life. Our data provide evidence that maternal STH infections predispose children to infections with STH parasites, and this effect was associated with elevated levels of IL-10 in newborn blood.

## Introduction

Soil-transmitted helminths (STHs, also known as geohelminths or intestinal helminths) are estimated to infect 2 billion people worldwide [1]. These parasites, of which *Ascaris lumbricoides* and *Trichuris trichiura* are the most prevalent, are a major cause of morbidity related to malnutrition and reductions in childhood growth [2]. In addition, STHs are considered to have potent immune modulatory effects, and may have an impact on the epidemiological distribution of other diseases. For example, *A. lumbricoides* and *T. trichiura* infections may contribute to an accelerated progression of HIV [3], a greater susceptibility to tuberculosis [4], and modify the development of allergy [1].

We still do not understand all of the factors involved in susceptibility to STH infections or the causes of the characteristic overdispersed or aggregated distributions of parasites [5]. Heterogeneity in exposure to infective stages may be relevant, but host factors must also be involved, as suggested by individuals re-acquiring heavy infections after treatment [5]. Understanding these factors is likely to help us develop novel interventions to complement mass drug administration (MDA) with anthelmintic drugs, and make MDA more efficient thereby reducing rates of re-infection.

Maternal infection with STH – and *in-utero* programming of the fetal immune response – may be one factor that contributes to disparities in infection outcomes. Previous studies have provided evidence that maternal infections with filiarial and schistosome helminths may affect the development of immune responses and susceptibility to infection during childhood [6], [7], [8].

Evidence for effects of maternal infection with STH on susceptibility to STH infections in offspring, in comparison, is more limited. A swine model of ascarid infection suggests that maternal infections affect susceptibility and parasite burden in piglets [9]. Additionally, it has been shown that exposure to maternal ascariasis is associated with evidence of immunologic sensitization to *Ascaris* antigens in newborn humans [10]. To our knowledge, there is no published evidence that maternal infections with STH have an effect on susceptibility to infection during childhood or on the modulation of systemic immune responses in childhood, despite the high prevalence of these parasites worldwide.

To determine if maternal infections with *A. lumbricoides* and/or *T. trichiura* affect susceptibility or intensities of infection with these parasites in early childhood, we compared parasitological data from children of uninfected and infected mothers to three years of age in a case-control study nested in an ongoing birth cohort study. We also looked for effects of maternal infection on induction of immune tolerance by measuring levels of IL-10 in newborn plasma.

## Methods

### Study population and subject recruitment

Mothers and their children were selected from the ECUAVIDA birth cohort study, an ongoing birth cohort study in the rural District of Quinindé in Esmeraldas Province, Ecuador. All pregnancies were uncomplicated vaginal deliveries, and follow-up evaluations including the collection of single stool samples were done at 3, 7, 13, 18, 24, 30, and 36 months. A detailed description of the aims and methods of this study is described elsewhere [11].

### Study design

The design was case-control nested in a birth cohort. Cases were defined as children between 7 months and 3 years of age with *A. lumbricoides* and/or *T. trichiura* infection. Only these two STH infections were considered because the prevalence of hookworm and *Strongyloides stercoralis* was less than 2% among the children in our study, and thus these data were unlikely to give meaningful results. Controls were defined as children between 7 months and 3 years of age with no STH infection. Exposure was defined as the presence of maternal infection with *A. lumbricoides* and/or *T. trichiura* detected in a stool sample collected in the third trimester of pregnancy. Inclusion into the study required data collected from stool samples. Stool samples were examined using the modified Kato-Katz and/or formol-ether concentration methods [12]. All cases and controls were selected from households where there was a previously documented infection with *A. lumbricoides* or *T. trichiura* determined by examination of stool samples collected within two weeks of birth from all household members. Households without a documented infection with *A. lumbricoides* or *T. trichiura* were excluded from the analysis because of probable limited risk of infection in such children. At the time this study was conducted, there was no program in place for the treatment of STH infections or malaria during pregnancy.

### Cytokine measurements

IL-10 levels in plasma from cord blood were measured in 90 individuals, 30 cases and 60 controls. Cases were children who had an infection with STH at 13–18 months of age while controls were children with no STH infection before 13–18 months of age. Maternal infection data had previously been collected during the third trimester of pregnancy for all cases and controls. IL-10 was measured in undiluted plasma, stored at −70°C using a commercial ELISA assay (Invitrogen) following the manufacturer's instructions. The limit of detection for this assay is 0.2 pg/ml.

### Statistical analysis

We estimated that a sample of 400 each of cases and controls would allow us to detect an OR of 1.5 with a maternal STH prevalence of 35%, an α of 0.05, and a power of 80%. For the purposes of analysis, three parasitological outcomes were considered: 1) susceptibility to STH infection – defined by a documented infection with *A. lumbricoides* or *T. trichiura* during the first 3 years of life; 2) susceptibility to high STH parasite burdens (defined by WHO-recommended thresholds of >50,000 eggs per gram [epg] of stool for *A. lumbricoides* and >10,000 epg for *T. trichiura* [13]) – based on egg counts of *A. lumbricoides* and *T. trichiura* from a stool sample collected at 3 years of age; and 3) aggregation – defined by the degree to which the estimated negative binomial parameter *k* tends to zero at 3 years of age for each of *A. lumbricoides* and *T. trichiura*. To evaluate differences in susceptibility between children of infected and uninfected mothers, we used logistic regression with adjustment for age, gender, maternal educational level, monthly income, number of household electric appliances, crowding (defined as number of persons per bedroom), bathroom facility, and water source. We also adjusted for the number of stool samples collected given that the more stool samples examined, the more likely an infection would be detected. All variables were included in the final model because precision of the estimated odds ratios did not change after removal of non-significant confounders [14]. The primary analysis examined effects of any maternal infection with *A. lumbricoides* and or *T. trichiura* on childhood susceptibility to infection with any STH infection (defined by an infection with *A. lumbricoides* and or *T. trichiura*). Secondary analyses looked at: 1) parasite-specific effects (*A. lumbricoides* vs. *T. trichiura*) on childhood susceptibility to any STH infection; 2) the effects of maternal co-infections on childhood susceptibility to any STH infection.

Intensities of infection with *A. lumbricoides* and *T. trichiura* were recorded as eggs per gram of stool. Stool egg counts were overdispersed (*A. lumbricoides*, mean 2,832 epg, variance 1.5×10^8^; *T. trichiura*, mean 317 epg, variance 4.8×10^6^), so a negative binomial model was used to fit the data, and potential confounders were controlled in multivariate analysis. The parameter *k* was also estimated and reported with Wald confidence intervals.

For analysis of cord blood levels of IL-10, two associations were evaluated: 1) the relationship between IL-10 and maternal infection status; and 2) the relationship between IL-10 and childhood infection status among children at 13–18 months of age. Geometric means were calculated, and Mann-Whitney U tests were used to compare IL-10 levels between groups.

All analyses were done in STATA (version 10.0) or in R (version 2.4.1).

### Ethics

The ECUAVIDA study protocol was approved by the Ethical Committees of the Hospital Pedro Vicente Maldonado and the Universidad San Francisco de Quito. Appropriate treatment was given to each individual with positive stool tests for STH infections as required by the study protocol.

## Results

### Population characteristics

Data from 1,004 mother-child pairs (410 cases and 594 controls) were included in the analysis out of a total of 1888 mother-child pairs with stool data collected to at least 7 months of age for each child eligible (884 pairs were excluded because of no documented infection with *A. lumbricoides* and *T. trichiura* among family members). Of the mothers included in the analysis, 23.6% (n = 237) were infected only with *A. lumbricoides*, 22.7% (n = 228) were infected only with *T. trichiura*, 18.6% (n = 187) were co-infected, and 35.1% (n = 352) were not infected. Geometric mean infection intensities with ascariasis and trichuriasis among all mothers were 7 (range: 0–130,970) and 5 (range: 0–20,510), respectively. The proportion of cases (38%) and controls (32%) with at least one infected household member was not significantly different. Summary statistics for cases and controls are provided in Table 1.

**Table 1:** Characteristics of cases and controls.

Characteristic	Cases (n = 410)	Controls (n = 594)
Child gender (male/female)	221/189	305/289
Mean child age at latest available checkup (months)	30 (13–51)	24 (7–44)
Monthly family income (US$)	158 (20–1000)	185 (30–1400)
Maternal educational level^a^	3 (1–7)	4 (1–7)
Crowding (occupants/bedroom)	3.7 (0.8–18)	3.0 (0.8–13)
Number of household electric appliances	2 (0–4)	3 (0–4)
Water sources		
Potable	33.9% (n = 139)	30.6% (n = 182)
Piped	11.5% (n = 47)	10.9% (n = 65)
Well	45.4% (n = 186)	52.5% (n = 312)
River, stream, or rain	5.1% (n = 21)	2.9% (n = 17)
Potable & well	1.5% (n = 6)	1.7% (n = 10)
Other combinations	2.7% (n = 11)	1.3% (n = 8)
Bathroom facility		
Latrine	75.9% (n = 311)	71.7% (n = 426)
WC	19.5% (n = 80)	25.9% (n = 154)
Field	4.6% (n = 19)	2.4% (n = 14)
Number of checkups	4 (1–6)	3 (1–6)
Childhood infection status		
Either	100% (n = 410)	0%
Single infections with *A. lumbricoides*	19.9% (n = 200)	0%
Infection intensity (epg)	5 (0–124,740)	0 (0–0)
Single infections with *T. trichiura*	7.0% (n = 70)	0%
Infection intensity (epg)	4 (0–30,310)	0 (0–0)
Both	23.6% (n = 140)	0%

Cases are children infected with either *A. lumbricoides* or *T. trichiura*, and controls are uninfected children. Values are frequencies (separated by/), percentages (%), and medians (range in parentheses). epg – eggs per gram of stool.

a 1 = illiterate, 2 = primary incomplete, 3 = primary, 4 = secondary incomplete, 5 = secondary, 6 = advanced incomplete, 7 = advanced.

### Relationship between maternal infection and childhood infection status

Children whose mothers were infected with STHs were 2.61-times more likely to be infected with STHs than children whose mothers were not infected (Table 2). Effects of maternal ascariasis and trichuriasis infection on childhood susceptibility to any STH infection were similar. Unique maternal ascariasis (i.e. in the absence of *T. trichiura*) raised the risk of STH infection in children by 2.19-fold (95% CI: 1.46–3.29, p<0.001). Likewise, unique maternal trichuriasis raised the risk of STH infection for children by 1.89-fold (95% CI: 1.26–2.81, p = 0.002).

**Table 2:** Effects of any, both, or single maternal infection with *A. lumbricoides* and *T. trichiura* on childhood susceptibility to any STH infection.

Infection of mother	No. of children infected^b^/total (% infected)	Univariate Analysis		Multivariate Analysis^a^	
		Odds Ratio (95% CI)	p	Odds Ratio (95% CI)	p
Any infection^b^	302/652 (46.3)	1.94 (1.47–2.59)	<0.001	2.61 (1.88–3.63)	<0.001
Co-infection	117/187 (62.6)	3.78 (2.56–5.58)	<0.001	5.91 (3.55–9.81)	<0.001
Single Infection^c^	185/465 (39.8)	1.49 (1.10–2.02)	0.007	2.08 (1.47–2.94)	<0.001
No	108/352 (30.7)	1.00		1.00	

a ORs adjusted for age, sex, maternal educational level, monthly income, household electric appliances, water source, bathroom facility, crowding, and number of follow-up evaluations.

b With *A. lumbricoides* and/or *T. trichiura*.

c With *A. lumbricoides* or *T. trichiura* only.

Abbreviations: CI = Confidence Interval.

Statistical comparisons were performed using logistic regressions.

### Effects of maternal co-infection on childhood infection status

The risk of childhood STH infection was even greater when mothers were co-infected. Children whose mothers were co-infected had 5.91-fold (adjusted 95% CI: 3.55–9.81, p<0.001) increased risk of being infected with STHs than children whose mothers had no STH infection. This is compared to a 2.08-fold increased risk for children of mothers with single infections (i.e. either *A. lumbricoides* or *T. trichiura* only) (Table 2).

### Relationship between paternal infection and childhood infection status

There seemed to be little association between paternal infection and childhood infection using data available from 531 fathers. No evidence was found for an association between paternal STH infections and child STH infections. There was also no evidence for associations between paternal infection with *A. lumbricoides* or *T. trichiura* and childhood susceptibility to general STH infection. There was, however, a significant relationship between paternal co-infection and childhood infection outcomes. Children of co-infected fathers were 2.54-fold more likely to be infected with STHs (95% CI: 1.25–5.19, p = 0.01).

### Effects of maternal infection on intensities and distributions

No significant relationship was observed between maternal infection and childhood infection intensity after adjusting for confounding factors (Table 3). Univariate estimates for *T. trichiura* were unreliable because of low egg counts - thus the wide confidence interval.

**Table 3:** Effects of maternal infection on childhood infection intensities and overdispersion.

	Maternal Exposure (n = 209)	No Maternal exposure (n = 155)	Parameter of overdispersion (*k*)		Univariate Analysis		Multivariate Analysis	
			Exposed (Wald CI)	Unexposed (Wald CI)	RR^a^ (95% CI)	p	RR (95% CI)	p
*A.lumbri-coides*	2,583 epg	3,168 epg	0.024 (0.016–0.030)	0.018 (0.011–0.025)	0.81 (0.19–3.41)	0.78	0.56 (0.11–2.95)	0.49
*T. trichiura*	490 epg	83 epg	0.031 (0.022–0.040)	0.031 (0.019–0.043)	5.90 (1.81–19.22)	0.003	1.26 (0.31–5.17)	0.78

a Rate Ratios (RR) calculated by negative binomial regression models and adjusted for sex, maternal educational level, monthly income, household electric appliances, water source, bathroom facility, crowding, and number of follow-up evaluations.

Abbreviations: CI = Confidence Interval.

Parameters were estimated and statistical comparisons were performed using negative binomial distributions.

### Systemic IL-10 levels in cord plasma

**Figure 1:**
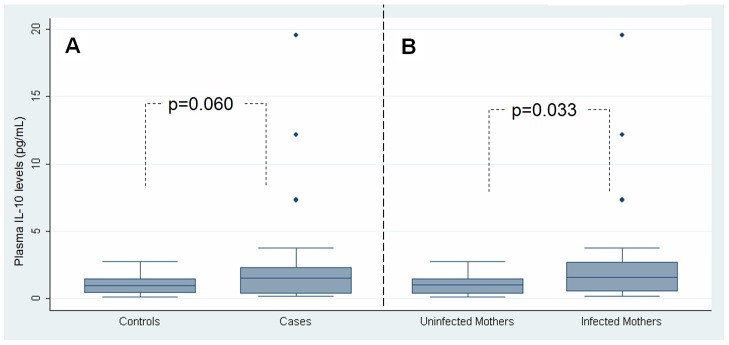
Plasma IL-10 levels in cord blood samples from newborns. Panel A: comparison of IL-10 levels between infected children (cases) and uninfected children (controls). Panel B: comparison IL-10 levels between children of uninfected and infected mothers. Box plots show median values (central line), inter-quartile range (box margins), 95% confidence intervals (bars), and outlying values (diamonds). Statistical comparisons were conducted using Mann-Whitney U tests.

Significantly greater levels of plasma IL-10 were also observed in cord blood from newborns of infected compared to uninfected mothers (geometric means: 1.32 pg/mL vs. 0.75 pg/mL, p = 0.033) (Figure 1). Subjects infected with *A. lumbricoides* or *T. trichiura* in later childhood (13–18 months of age) had a trend of greater levels of IL-10 in cord blood plasma than children who were not infected between 13–18 months of age (geometric means: 1.20 pg/mL vs. 0.77 pg/mL, p = 0.060). Interestingly, two of three outlying values were from co-infected mothers with the highest infection intensities. Outliers did not affect the non-parametric analysis used.

## Discussion

This study provides evidence that children whose mothers are infected with the STH parasites, *A. lumbricoides* and *T. trichiura*, have an increased risk of infection compared to children of uninfected mothers. This increased risk was most pronounced for children of mothers infected with both parasites. We did not observe an effect of maternal STH infection on the intensities of infection or distributions of parasites in offspring– an observation that likely reflects the relatively low parasitic infection intensities observed at 3 years of age. It is important to remember that STHs do not multiply within the host, but instead are acquired over time by continual exposure to embryonated eggs in the environment [15].

We believe that the observed increase in susceptibility could be the result of *in utero* immune modulation that induces greater neonatal and childhood tolerance. This is predicated upon findings that maternal ascariasis and trichuriasis alter newborn Th1 and Th2 anti-parasite cytokine responses [10]. Furthermore, there is strong evidence of *in utero* sensitization and induced tolerance to other helminths [7], [8]. The precise mechanism by which the fetus is sensitized is unclear, but helminth antigen may cross the placenta because *Ascaris* antigen has been found in peripheral blood [16]. Trans-placental infection seems highly unlikely because we found no eggs in stool samples collected at 3 months of age. Further, to our knowledge there is no published evidence showing that *A. lumbricoides* and *T. trichiura* infections can be transmitted *in utero*.

We hypothesized that the increased susceptibility to infection through having an infected mother could be mediated by IL-10, an immune modulatory cytokine that is produced by regulatory cells [21]. Increased production of IL-10 is a feature of chronic STH infections and is reflected in elevated plasma levels [11] and greater constitutive production by peripheral blood leukocytes (PBLs) [17], [18], [19], [20]. The treatment of ascariasis has been associated with a decline in levels of plasma IL-10 [11]. Similarly, children of mothers infected with filarial parasites have an increased susceptibility to filarial infection, their PBLs produce elevated levels of IL-10 constitutively [6], and they show impaired T-cell responses [8].

Our observation of elevated levels of IL-10 in cord blood plasma of the infants of infected mothers and in cord blood plasma of infected children provides a potential mechanism by which maternal infections may subsequently increase susceptibility to infection in children. Elevated levels of IL-10 may have important effects in the modulation of inflammatory responses to the parasite that prevent potentially damaging pathology but increase susceptibility to infection [18]. Such non-specific effects on the host systemic immune response would be predicted to have effects on other inflammatory conditions such as allergy in which IL-10 is considered to have an important modulatory role [21]. In support are observations from recent studies that indicate that maternal STH infections may protect against infantile eczema [22] and modify immune responses to tuberculin [23].

There is a complex relationship between exposure to parasites and the resulting distributions of parasites and susceptibility to infection among hosts, so there may be other explanations for household clustering of infection other than *in utero* antigen exposure. For example, the observed maternal effect may have been a product of residual confounding (i.e. that which is not removed or accounted for by our analysis). To minimize such a bias, our study attempted to control for demographic, socioeconomic, geographic, and behavioral risk factors. Our inclusion criteria, for one, required a documented infection in at least one family member at the time of the child's birth to try to control for the risk of exposure. Moreover, in the analysis we adjusted for six potential confounding variables that are closely linked to the presence of helminth infections [24]. Three of these – crowding, bathroom facility, and water source – are behaviorally related, and have been previously found to be risk factors for STH infections in pregnancy and in infants [25], [26].

Alternatively, children of infected mothers may have been genetically predisposed to STH infection as evidence has been found for a genetic contribution toward susceptibility [27], [28], [29]. However, the weak association between paternal and childhood infection status argues against this explanation and is consistent with findings from previous studies that have shown weak or no associations between paternal helminth infection and childhood susceptibility to infection [6], [30]. Finally, we cannot exclude the possibility that light infections were missed although we employed a combination of parasitologic methods to maximize sensitivity.

In conclusion, our data provide evidence that maternal infections with the STH parasites, *A. lumbricoides* and *T. trichiura*, are associated with an increased susceptibility to infection in children during the first 3 years of life. One possible mechanism by which this effect may be mediated is plasma IL-10 that was elevated in cord plasma of newborns of infected mothers and in cord plasma of children who later became infected with STHs. If maternal infection does induce long-lasting tolerance in children and such an effect is associated with elevated plasma IL-10 from birth, our data may provide an explanation for the apparent protective effect of maternal helminth infections against childhood allergy and their possible deleterious effects on other infectious diseases such as HIV, TB, and malaria [22], [23], [31].
